# The Use of Photoactive Polymeric Nanoparticles and Nanofibers to Generate a Photodynamic-Mediated Antimicrobial Effect, with a Special Emphasis on Chronic Wounds

**DOI:** 10.3390/pharmaceutics16020229

**Published:** 2024-02-05

**Authors:** Mohamed A. Abdel Khalek, Amr M. Abdelhameed, Sara A. Abdel Gaber

**Affiliations:** 1 Institute of Nanoscience and Nanotechnology, Kafrelsheikh University, Kafrelsheikh 33516, Egypt; 2 Institute of Global Health and Human Ecology, School of Sciences & Engineering, The American University in Cairo, Cairo 11385, Egypt; 3 Bioscience Research Laboratories Department, MARC for Medical Services and Scientific Research, Giza 11716, Egypt; 4 Nanomedicine Department, Institute of Nanoscience and Nanotechnology, Kafrelsheikh University, Kafrelsheikh 33516, Egypt

**Keywords:** chronic wound healing, polymeric nanoparticles, electrospun nanofibers, photodynamic therapy (PDT)

## Abstract

This review is concerned with chronic wounds, with an emphasis on biofilm and its complicated management process. The basics of antimicrobial photodynamic therapy (PDT) and its underlying mechanisms for microbial eradication are presented. Intrinsically active nanocarriers (polydopamine NPs, chitosan NPs, and polymeric micelles) that can further potentiate the antimicrobial photodynamic effect are discussed. This review also delves into the role of photoactive electrospun nanofibers, either in their eluting or non-eluting mode of action, in microbial eradication and accelerating the healing of wounds. Synergic strategies to augment the PDT-mediated effect of photoactive nanofibers are reviewed.

## 1. Chronic Wounds and the Role of Biofilm

Chronic wounds are defined as wounds that take from one month to three months or more to heal. There are various types of chronic wounds, such as, venous ulcers, arterial ulcers, pressure ulcers, surgical wounds, radiation wounds, and diabetic foot ulcers (DFUs) [1]. Generally, older adults are at the highest risk for the development of chronic wounds due to multifactorial geriatric syndrome and age-related diseases such as hypertension, diabetes, and dyslipidemia [2]. After an injury, the body initiates the hemostasis phase to stop blood loss by constricting blood vessels and forming clots. In the following inflammatory phase, phagocytes exert an important role. Neutrophils secrete reactive oxygen species (ROS) and proteases, which prevent microbial invasion and remove debris. Monocytes differentiate into macrophages that have strong microbial phagocytic actions and release cytokines that attract fibroblasts, keratinocytes, and endothelial cells for tissue repair. The proliferation phase begins after the immune cells undergo apoptosis, and granulation tissue, blood vessels, and epithelialization are formed. Finally, the remodeling phase takes more than a year to complete [3].

Chronic wounds are often unable to proceed through the inflammatory phase due to an excessive generation of ROS, inflammatory cytokines, and metalloproteinase. The interplay between these factors is complex, leading to a continuous degradation of the extracellular matrix. This unstable matrix makes it difficult for healing cells to anchor themselves, and, as a result, they lose their ability to function properly [4]. DFUs present a significant economic and health issue, with high morbidity and mortality rates. Globally, around 6.2% of diabetic patients suffer from ulceration, and it is estimated that to cure one diabetic foot patient, USD 8659 will be spent on a yearly basis [5]. These wounds are hard to heal due to their altered immune state, neuronal response, and microvascular blood supply [6]. Furthermore, these factors may trigger chronic microbial infections, creating poor prognostic conditions that can finally lead to limb amputation [7]. The management of a DFU is considered a multidisciplinary intervention, prompting accurate assessment of the ulcer grade and degree of microbial invasion [8]. Generally, wound offloading, ulcer debridement followed by disinfection, is crucial for the removal of necrotic tissue and microbial flora [9]. Choosing the appropriate dressing type based on ulcer conditions is crucial to achieve a moist wound bed, protection against microbial invasion, a high absorptivity of exudates, and cost-effectiveness. A plethora of other interventions are adopted, for instance, topical and systemic antimicrobials [10], cellular bio-products, skin grafts [11], human growth factors [12], and energy-based therapies [13].

The chronicity of wounds in diabetes is caused by a combination of long-term hyperglycemia, altered immune responses, reduced blood supply, and microbial biofilm formation [14]. The severity of biofilms arises from their ability to form a protective barrier. In essence, a biofilm is a community of microbial cells that attach to non-living or living surfaces and produce an extracellular matrix consisting of polysaccharides, proteins, lipids, and nucleic acids. This matrix shields the bacterial cells from antimicrobial agents and the host’s immune system, providing them with a haven to thrive and grow [15]. Polysaccharides (PLSs) are considered an integral part of the exopolymeric structure of a biofilm. Indeed, PLSs play a key role in biofilm establishment during the whole biofilm cycle, which ranges from surface attachment to disassembly and release [16]. Extracellular matrix proteins also play a pivotal role in biofilm establishment and maintenance. They have various functions, such as their involvement with cells at surface attachments. Moreover, they are involved in biofilm stabilization by their attachment to PLSs and extracellular nucleic acids within the biofilm mass. Cell wall anchoring proteins, for example, exert strong biofilm stabilizing activity in *Staphylococcus aureus* (*S. aureus*) [17]. The enzymatic role of proteins is needed for the degradation of PLSs, proteins, and extracellular nucleic acids. Extracellular DNA also may play a critical role in biofilm architecture; moreover, it can grant the biofilm great resistance to antimicrobials [18].

Quorum sensing, on the other hand, represents the fundamental machinery for cell-to-cell communications [19]. Microbial cells can produce signaling molecules called ‘autoinducers’ depending on cells’ density in the biofilm. These chemical entities are considered genetic expression modulators; they can regulate the transcription of certain genes responsible for granting resistance to the biofilm mass against environmental conditions, host defense, and antimicrobials. Homoserine lactones, for example, are considered the dominant Gram-negative autoinducers [20]. The close contact of microbial cells imposes flexibility in transferring genetic materials such as plasmids between cells [21]. Additionally, the deeply embedded microbial cells become dormant due to a lack of nutrients and oxygen, thus they exhibit higher antimicrobial resistance than superficial cells [22]. In brief, the co-existence of the biofilm components can trigger severe inflammatory responses; subsequently, this will lead to the accumulation of neutrophils, metalloproteinase, and other inflammatory mediators, triggering the chronicity of the ulcers [15].

Tissue revitalization through sharp or surgical debridement is considered crucial in eradicating biofilms from wound beds [23]. There are other physical methods, such as electrical and ultrasonic techniques [24]. Direct chemical methods are utilized for biofilm eradication, which lead to the uncovering of deeply embedded recalcitrant cells. Furthermore, indirect interventions such as inhibiting cell attachment, altering cell-to-cell signaling, and altering cell metabolism are potential targets for further investigation [25]. Lactoferrin, for example, has been investigated in biofilm dispersal through its iron chelation and DNAse activity [26]. Nitric oxide is thought to be active through the intracellular messenger di-GMP, initiating a range of effector functions for biofilm disruption [27].

## 2. Challenges and Innovations in Dealing with Chronic Wounds and Biofilms

The handling of chronic wounds faces major obstacles that hinder the success of treatment protocols. One of the challenges is precise diagnosis. The most common types of chronic wounds are ulcers, ischemic wounds, and infectious ones. The physical assessment is combined with laboratory assessments. The German Wound Association developed a diagnostic rule, abbreviated as ABCDE, where anamnesis, bacterial identification, clinical examination, defective vascular system, and extra assays are conducted [28]. Failure to properly identify the etiology of the wound and the involved microorganism species and strains complicates the treatment process and aids in the development of resistant strains. One of the common mistakes is to collect swabs of all wounds and prioritize that over the physical examination. It is imperative to bear in mind that all wounds are contaminated with microorganisms, yet their deleterious impacts are not caused by the whole set of those microorganisms [29]. Another major challenge is treating the biofilm that is present in 90% of chronic wounds, which is polymicrobial in nature. Biofilms vary in their metabolism, distribution, composition of extracellular polymeric substances that can be used for targeting, and microenvironment composition, which results in their differential responses to therapy. Due to their genomic instability and developed inter-species interactions, treatment protocols cannot be generalized [30]. A complete destruction of the biofilm requires the combination of antiseptics and debridement protocols. However, the use of local or systemic antibiotics is not accurately decided [31]. While numerous strategies are implemented and a plethora of scientific studies promise a better management of chronic wounds, clinically, the status of chronic wound management is below satisfactory. It all revolves around the necessity of patient–care center interactions on a frequent basis for a long duration. Unlike other ailments, healing chronic wounds and eradicating bacterial biofilms demand repetitive visits for a duration that may be as long as a year in some cases. This review [32] discusses the underlying reasons for this from a pharmacoeconomic point of view. Pathologically, there are numerous key players that impair wound healing, since proper wound healing requires an orchestrated performance from multiple components of the immune system. An overview of this highly dynamic and complex process that depicts the main components is provided in this review [33].

In severe cases, skin substitutes are added to boost the wound healing process in advanced cases of chronic wounds such as DFUs. Commercially, acellular and cellular skin substitutes exist, and new ones are expected to emerge on the market soon as there are many ongoing clinical trials examining the efficiency of new substitutes. Nevertheless, limited information is known about clinical and patient outcomes after the end of treatment [34]. Emerging technologies such as non-coding RNA [35], smart dressing supplemented with monitoring sensors for progress follow-up [36], and microbiota [37] are just examples of modalities that might revolutionize the management of chronic wounds.

Photodynamic therapy (PDT) is a simple and promising local therapy for chronic wounds [38] and microbial eradication either in planktonic or biofilm patterns [39,40]. In a recent systematic review, PDT studies involving animal models of wound healing were presented. A reduction in wound size and decline in bacterial counts within the wound bed, along with an elevation of healing cytokines such as β-fibroblast growth factor, vascular endothelial growth factor, and many other growth factors, were reported [41].

## 3. Basic and Underlying Mechanisms of Photodynamic Therapy (PDT)

**Figure 1:**
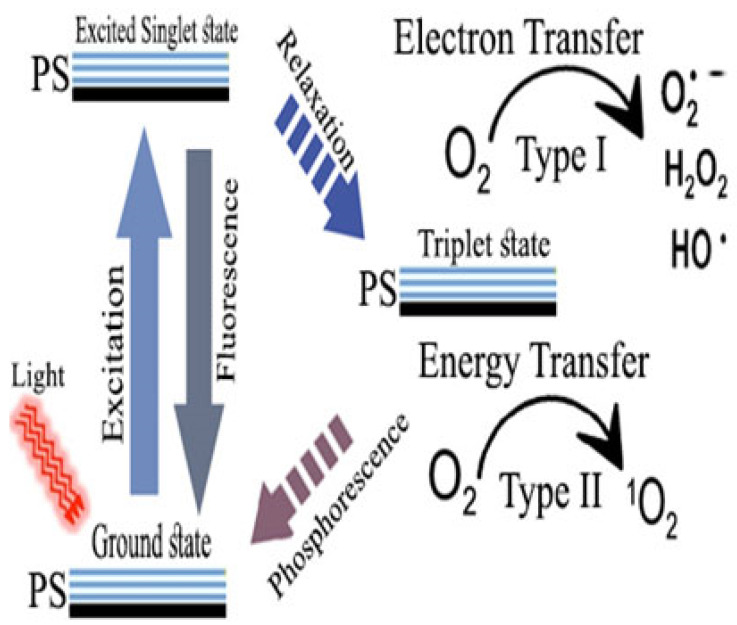
Schematic presentation of photodynamic therapy (PDT). When light at specific wavelengths is applied to the PS molecules, they are excited to a high-energy singlet state, which can then be relaxed to the longer-life triplet state via intersystem crossing. This process can result in either type I or type II reactions.

The foundation of PDT is that, in the presence of oxygen, a light-responsive compound known as photosensitizer (PS) undergoes a photochemical process that can follow either type I or type II reactions (Figure 1). Recently, a type III reaction was introduced, in which the PS binds to RNA and, in this way, the excited PS transfers its energy to RNA, bypassing the need for oxygen, thus it is well suited to deep hypoxic regions [42]. Numerous sources of light currently exist, as summarized in this review [43]. PSs share key characteristics, namely excitability upon light irradiation of a certain wavelength, photostability, good quantum yield, and the ability stay for a long duration in the triplet state. In terms of developmental stages, PSs are classified into first-, second-, and third-generation PSs. While some of the first-generation PSs are clinically approved, such as Photofrin, more of the second-generation PS are in use and commercially available, such as 5-aminolevulinic acid and temoporfin. The third-generation PSs include targeted PSs using antibodies, for instance, in addition to nano-formulated PSs [44]. A more common classification of PSs relies on the mechanism of their activities. In this regard, they are classified as Type I or Type II PSs, such as the phenothiazine family. In type I PSs, the excited PS donates hydrogen to the nearby carbohydrate, protein, or lipid, while, in Type II, oxygen is the recipient and a series of ROS such as •OH, H_2_O_2_, and O_2_^•−^ are generated [44].

The clinically used PSs include methylene blue (MB), which is a hydrophilic phenothiazine derivative and thus a water-soluble compound. It is stimulated using 635 nm and known for its broad-spectrum antimicrobial activity [45]. MB is intensively studied in periodontal infections thanks to its inert nature and need for short irradiation times to become excited [46]. Chlorin derivatives such as Temoporfin, which is commercially known as Foscan^®^, are widely applied. It is excited using 652 nm and tends to accumulate in the mitochondria [47]. The list of PSs is expanding thanks to the ongoing research at the clinical level and the synthesis of new derivatives.

**Figure 2:**
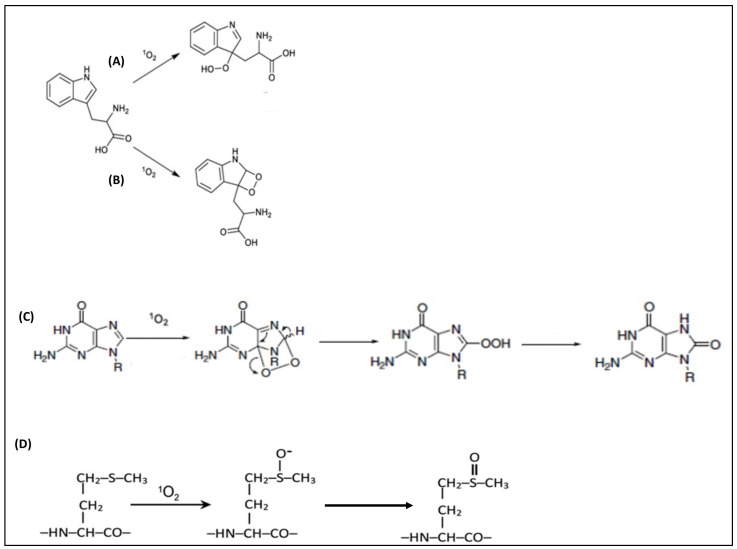
Schematic presentation of singlet oxygen attacking electron-rich centers: (**A**) ENE reaction, (**B**) (2 + 2) cycloaddition reaction, (**C**) (4 + 2) cycloaddition reaction, and (**D**) reaction with sulfur-containing amino acids [51,52,53].

For biofilm eradication via PDT, generated ROS attacks target biomolecules such as aromatic- and sulfur-containing amino acids. Furthermore, unsaturated fatty acids and bases of the nucleic acids are common targets for ROS. These biomolecules are abundant in the biofilm extracellular matrix or within the cell membrane and the protoplasm of microbial cells. Among the ROS, singlet oxygen (^1^O_2_) has relatively specific interactions with its target. It exhibits four fundamental reactions depending on the target moieties. They are the ENE reaction, (4 + 2) cycloaddition reaction, (2 + 2) cycloaddition reaction, and the attacking of electron-rich heteroatoms, such as the sulfur atom of methionine, as illustrated in Figure 2. It is worth noting that the previously described mechanisms can overlap sequentially [48].

At the cellular level, responses are clustered as either vascular cessation, immunostimulation, or the induction of a cell death mode, namely apoptosis or necrosis. There is no specific order or an exclusive response to a particular PS. An interplay between these three responses takes place and, collectively, they mediate the observed therapeutic response [49,50].

## 4. Major Limitations of PDT

The non-specificity of the target is a double-edged sword for PDT. On one side, having a versatile application of the same treatment modality is economically advantageous since the same PS can be used to treat a wide spectrum of pathogens [54]. In fact, in our studies, we could use the same PS, which is chlorin e6 (Ce6), for anticancer and antifungal activities [55,56,57]. Furthermore, this implies that no or minimal resistance is developed, which is lifesaving, as the globe is facing a massive case of resistance to standard antibiotics because of the abusive consumption of the available ones. On the other hand, the absence of a specific target renders the standardization of PDT protocols unproductive. It is rather a customized protocol with general and very broad guidelines.

For a long time, PDT was restricted to topical applications, where illumination is very accessible. That can be seen by the frequent studies and granted approval of 5-aminolevulinic acid for the treatment of actinic keratosis [58]. This suits the use of PDT for chronic wound healing, since injured skin regions are easily accessible. In a recent study, 5-aminolevulinic acid PDT was tested for its ability to eradicate biofilm-forming bacteria using the Lubbock chronic wound biofilm model. The model consisted of resistant strains of *Pseudomonas aeruginosa* (*P. aeruginosa*) and *S. aureus*. Combining PDT with graphene oxide (GO) nanoparticles resulted in a 90% reduction in CFU/mg that could be explained by the elevated levels of ROS generated [59]. Yet, with the advances in photonics, internal applications of PDT such as in the treatment of non-small lung cancer have been approved by Photofrin [60].

Many PSs are chlorophyll- and xanthene-based compounds. Additionally, many natural compounds such as curcumin [61] and hypericin are employed as PSs [62]. This natural origin explains their wide safety window. However, their low hydrophilicity limits their bioavailability. Several solutions were proposed to overcome this limitation, and utilizing nanoparticles was one of the successful strategies that showed promising results at the clinical level [63]. A full spectrum of nanoparticles including metallic, polymeric, carbon-based, and nanosheets combined with PDT for skin, bone, and cartilage healing was presented in an earlier review [64] Throughout the four identified wound healing stages, nanoparticles such as ceramics, lipids, nanohydrogels, and nanocomposites, in addition to other forms loaded with PSs, showed a significant improvement in the wound healing outcome, as summarized earlier [65]. While nanoparticles are diverse, this review addresses, in particular, the polymeric nanoparticles and electrospun nanofibers that have been combined with PSs to enhance PDT-mediated chronic wound healing.

## 5. Polymeric Nanoparticles with Intrinsic Activity: Enhancing PDT and Their Role in Wound Healing

Polymeric nanoparticles are among the most heavily studied nanoforms for drug delivery. They are broadly classified into nanospheres and nanocapsules. The most famous examples of polymeric nanoparticles are liposomes, the first FDA-approved nanoform of which was Doxil. It is composed of pegylated liposomes loaded with doxorubicin [66,67]. Because of their physicochemical characteristics, liposomes keep the wounded sites moist and thus are employed as wound dressings [68]. Polymers used in the synthesis of polymeric nanoparticles are either natural, such as chitosan, synthetic, such as poly (lactide-co-glycolide) (PLGA), or, in many cases, a polyblend of polymers [69]. In a recent study, a poly (L-lactic acid) nanoparticle loaded with both curcumin and nisin was synthesized to release the drug in response to ultrasonic waves, known as sonodynamic therapy. The results demonstrated a continuous release for up to 14 days. By extending the duration of light irradiation, intensifying ultrasonography, and increasing the concentration of the nanoparticles, *Acinetobacter baumannii* (*A. baumannii*) cell viability was reduced in a dose-dependent manner. Mice showed a time-dependent decrease in biofilm formation, alterations in gene expression, and an acceleration of skin re-epithelialization significantly larger than the positive control group treated with silver sulfadiazine [70]. Lipid nanoparticles represent polymeric nanoparticles, known for their high entrapment efficiency of lipophilic drugs such as protoporphyrin. In a recent study, gallium protoporphyrin lipid nanoparticles demonstrated PDT properties against *S. aureus* biofilms. The synthesized lipid nanoparticles demonstrated enhanced antibacterial action, lowering bacterial viability significantly at the in vivo and ex vivo levels. Furthermore, by lowering the bacterial load and enhancing early collagen deposition, the synthesized lipid nanoparticles dramatically accelerated wound healing [71].

The following are selected examples of polymeric nanoparticles known for their intrinsic activities that boost PDT and their wound healing activities.

### 5.1. Polydopamine Nanoparticles (PDA)

Polydopamine nanoparticles (PDA) are widely used in smart formulations due to their intrinsic properties, such as photothermal conversion [72,73]. Additionally, the polyhydroxyl groups on their surface provide excellent sites for metal chelation, and these metals such as PDA-Cu and PDA-Ag exhibit strong antibacterial efficacy [74]. Their enriched double bond allows for satisfactory drug loading via π–π stacking; consequently, the hydrophobic and poorly water-soluble PSs can be effectively loaded into PDAs [75]. Loading drugs can occur either during the synthesis process of PDA or as a separate post-synthetic step, and the drug release can be stimulated by laser irradiation, or it may follow a passive route. In this way, PDA can be tailored, in its synthesis and properties, to be a drug delivery system [76]. It was shown that the encapsulation efficacy of Ce6 in mesoporous PDA nanoparticles reached around 69.64% [77]. These loadings may effectively inhibit PS aggregation, leading to a longer lifetime of the triplet state and a higher quantum yield of singlet oxygen. For instance, curcumin-loaded PDA exhibits a high quantum yield of singlet oxygen compared to free curcumin [78]. PDA’s ease of surface functionalization allows for the fabrication of smart microbial-selective PDT nanoparticles. PDA nanoparticles that are responsive to pH levels have been designed. This nanosystem consisted of the PS Rose Bengal (RB) loaded on PDA nanoparticles coated with polymyxin B and gluconic acid to create pH-responsive nanoparticles. When exposed to an acidic and infectious environment, the nanoparticles underwent a positive surface charge conversion that allowed for their efficient binding to the microbial membrane and increased their photo-inactivation efficiency against Gram-negative bacteria [79].

Besides the direct antibacterial effect generated from the PDA–metal chelates, the photothermal effect is enhanced by Ag chelation. A previous study documented that the covering of PDA with Ag was found to enhance the nanoparticles’ photothermal efficacy; additionally, the incorporation of cationic guar gum (CG) hydrogel prevents the aggregation of PDA@Ag. The fabricated CG/PDA@Ag exhibited a significantly higher photothermal conversion efficacy compared to PDA and, thus, enhanced antimicrobial efficacy [80]. A methicillin-resistant *Staphylococcus aureus* (MRSA) infection was successfully combated at the in vitro and in vivo levels using PDA-mediated photothermal therapy combined with Ce6-mediated PDT. The nanoconstruct consisted of magnetic iron oxide nanoparticles coated with a layer of polydopamine that enhanced their response to NIR. PDA acted as a linker to which Ce6 was covalently bound, and the whole system was coated with chitosan. The positive charge of chitosan facilitated a good interaction with the bacteria and its magnetic properties allowed for the isolation of bacteria in response to an external magnetic field [81]. The coating of selenium nanoparticles with polydopamine was recently reported as a nanoplatform for the PS indocyanine green. A total eradication of *S. aureus* and *Escherichia coli* (*E. coli*) was reported after 808 nm irradiation, and the system accelerated wound healing with a wound closure of 88% in only 8 days [82].

### 5.2. Chitosan Nanoparticles

The most significant chitin derivative is chitosan, which is produced when chitin’s acetate moiety is removed. It comes from the cell walls of fungus and crustacean shells, like those of prawns or crabs. It is a cationic, mucoadhesive, biocompatible polymer that occurs naturally and is licensed by the FDA for use in tissue engineering and medication delivery systems [83]. Studies have shown that chitosan can expedite the healing of skin wounds by stimulating the development of capillaries, fibroblasts, and inflammatory cells, which are typified by macrophages. Chitosan can stimulate macrophages to secrete wound healing cytokines such as transforming growth factor-β, platelet derived growth factor, and a series of interleukins [84]. Chitosan’s favorable qualities—such as its capacity to absorb water, absorb antibacterial activity, its biocompatibility, biodegradability, and function as a biological membrane—have led to its widespread usage as a wound dressing in recent years [85].

Chitosan nanoparticles are among the most widely used nanocarriers for drug delivery due to the abundance of chitosan polymers, the ease of fabrication of the nanoparticles, and, importantly, their intrinsic antimicrobial activity [86,87]. As a result, several studies have been performed to estimate the synergic effect of chitosan nanoparticles, as intrinsically active nanocarriers, with PDT.

It is known that chitosan nanoparticles exhibit strong biofilm disruption efficacy due to the polycationic structure of chitosan, consequently, it has been proposed that loaded PSs can reach the deeply embedded dormant microbial cells within an exopolymeric biofilm structure. Esmaeil Darabpour et al. documented that cationic phenothiazine PSs, such as MB, loaded in chitosan nanoparticles exhibited a strong effect against *S. aureus* and *P. aeruginosa* biofilms when compared to free MB [88]. Moreover, it was found that the PDT activity of non-cationic PSs such as Ce6 can be enhanced by their conjugation with polycationic chitosan nanoparticles. For instance, anionic Ce6 can be electrostatically assembled on the surface of the cationic coat of nanoparticles to reach deep biofilm components [89]. The free carboxylic group of Ce6 enabled its covalent attachment to the chitosan. These nanoparticles demonstrated PDT activity comparable to vancomycin against MRSA and *A. baumannii* [90]. It was reported also that chitosan nanoparticles augment the photo–sonodynamic effect of indocyanine green for polymicrobial biofilm destruction [91]. Light-responsive multifunctional nanoparticles were designed to combine Ce6 with quaternary ammonium chitosan and natural antimicrobials. When exposed to light at 660 nm, the Mg/(−)-epigallocatechin-3-gallate combination released magnesium ions quickly, which successfully sped up wound healing. Notably, an efficient binding to bacteria was reported, and the irreversible destruction of the bacteria’s membrane structure by ROS resulted in the death of the bacteria [92]. One of the key features of chitosan nanoparticles as a carrier system for PS is their augmented adsorption capacity. Silver nanoparticles were coated with bovine serum albumin and encapsulated in chitosan nanoparticles that allowed for MB adsorption. Adsorption was achieved via the electrostatic interaction between the negative chitosan and the positive MB molecules, in addition to the chelating force of chitosan. Thus, the developed system exhibited a potent antibacterial activity compared to its individual components [93]. Table 1 summarizes some of the recently conducted studies where chitosan nanoparticles synergized the antimicrobial activity of PDT.

### 5.3. Polymeric Micelles

Polymeric micelles can be easily tailored to allow for various physiochemical properties of the loaded drugs, including vaccines, as reviewed earlier [102,103]. Proper copolymer selection can improve the biofilm penetration efficacy of the PS [104,105]. Table 2 summarizes some of the recent studies in which micelles were employed for PS loading to mediate the inactivation of bacteria using PDT. For instance, the antibacterial and antibiofilm effects of curcumin-functionalized PLGA-dextran micelles were examined against *Pseudomonas putida* and *Pseudomonas fluorescens*. The presence of dextran, as the hydrophilic part of the amphiphilic polymer, was responsible for the remarkable antibiofilm effect observed. It allowed for good adherence and penetration across the exopolysaccharide matrix of the biofilm [106]. The amphiphilic copolymers with cationic moieties are also considered strong enhancers for the dissipation of the exopolymeric matrix of the biofilm with higher selectivity. Qiang Gao et al. designed a new PDT delivery system via the supramolecular assembly of Ce6-conjugated α-cyclodextrin and polyethylene glycol (PEG) polypeptide (Pep). These cationic Pep@Ce6 micelles were proven to be highly effective in biofilm disruption and deactivating recalcitrant microbial cells, with minimal toxicity to human cells [107].

Polymeric micelles can be designed to specifically bind to microorganisms by covalently conjugating pathogen-targeting peptides [108]. Additionally, the micelles can respond to the infected environment by designing amphiphilic copolymers that undergo charge reversal based on minor differences in pHs [109]. This allows for a more targeted and effective treatment of infections. Shuting Wang et al. conjugated PEG-b-poly(2-(hexamethyleneimino) ethyl methacrylate-co-aminoethyl methacrylate) with Ce6 to create pH-responsive prodrug micelles. The objective was to enhance the antimicrobial activity of PDT. The assembly was negatively charged under normal physiological conditions to ensure long blood circulation. However, in a bacterial infection microenvironment, the micelles became positively charged, allowing them to adhere to negatively charged bacterial membranes. This resulted in a significant improvement in their antibacterial activity against MRSA and *E. coli* after laser irradiation [110]. Another infection-targeted nano-micelle was designed that depends upon the charge reversal properties. This nanoform utilizes an ester bond to conjugate betaine carboxylate (CB) with ciprofloxacin. This micellar assembly encapsulates (5, 10, 15, 20-tetraphenyl porphyrin) as the PS. In an acidic pathogenic microenvironment, the betaine moiety becomes positively charged and binds tightly to the biofilm matrix; additionally, lipase triggers the release of ciprofloxacin and the PS [111].

**Table 2:** Summary of some studies on polymeric micelles of PSs to mediate antimicrobial PDT.

Nanosystem Structure	PS	Major Findings	Ref.
Self-assembled glycol chitosan micelles incorporating a PS	Protoporphyrin IX	- Positively charged micelles were developed. - The nanosystem disassembled upon interaction with the microbial cell membrane. - Microbial cell death involved damage to the DNA.	[112]
Lipase-sensitive methoxy PEG-block-PCL micelles	Hypocrellin	- Lipase was secreted upon interaction with the bacterial cell membrane and mediated the release of the PS. - The micelle improved the stability and water solubility of the PS. - Antibacterial activity against MRSA was confirmed at the in vitro and in vivo levels with good biocompatibility and no hemolytic activity.	[113]
Pluronic micelles of either P84, P123, or F127	Curcumin	- The lowest PS release rate was observed when P84 micelles were tested. - The produced micelles had a diameter range between 18 and 30 nm. - The irradiated formulations exerted a strong antibacterial activity against *E. coli*, *S. aureus*, and *C. albicans* and were inert to fibroblasts.	[114]
Self-assembled micelles of PEG-b-poly(2-(hexamethyleneimino) ethyl methacrylate-co-aminoethyl methacrylate)	Ce6	- The surface charge of the micelles changed from negative while circulating in the blood flow to positive when placed within a bacterial microenvironment. - MRSA and *E. coli* responded efficiently to the irradiated micelles in the isolated pathogenic form and at the in vivo level.	[110]
F-127 micelles	Safranine-O	- Inactivation of *E. coli* and *S. aureus* was achieved at lower minimum inhibitory concentrations compared to free PS.	[115]
Lauric arginate ethyl ester micelle	Curcumin	- Polymicrobial cultures of *E. coli* and *Listeria innocua* were inactivated upon exposure to UV-A light, as indicated by their leaked protein and DNA. - Synergy between PDT and antimicrobial activity of curcumin was documented. - The antimicrobial activity was pH-dependent and the best results were obtained at a pH of 7.	[116]
Poly (PEG)-block-poly (lactic acid) micelles	Tris(1,10-phenanthroline) ruthenium (II) bis (hexafluorophosphate)	- Singlet oxygen was produced upon illumination with blue light. - Planktonic Pseudomonas aeruginosa responded to PDT mediated by micelles 10 times more than it did to free PS.	[117]

## 6. Electrospun Nanofibers: Basics of Synthesis

Nanofibers are well suited to wound healing as their offered wide surface area allows for the covering of wounds, including large ones. There are several techniques available for fabricating nanofibers including self-assembly, phase separation, template synthesis, electrospinning, and blow spinning. Out of these, electrospun nanofibers are applied due to their ease of fabrication, the better control of the fiber diameter under electrospinning conditions, and versatility in the selection of polymers for nanofiber fabrication [118,119].

**Figure 3:**
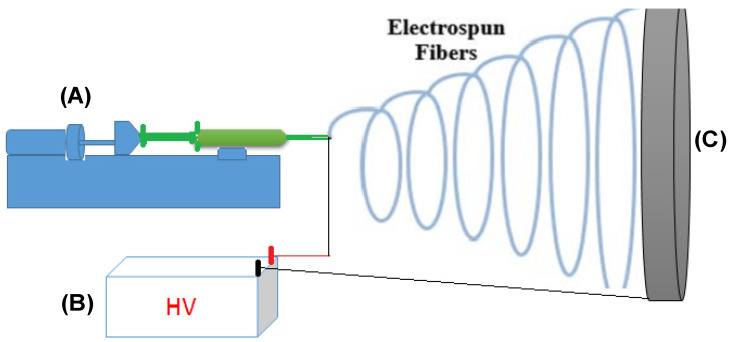
Schematic presentation of the basic setup of an electrospinning system, its (**A**) syringe pump, (**B**) high-voltage power supply, and (**C**) collector.

Electrospinning has been used for nanofiber production since the first patent for yarn production using this technique was filed in 1934. Despite the current complexity of commercially available electrospinning instruments, they all require the same basic components: a high-voltage power supply, a continuous polymer solution or polymer melts feeding system, and a collecting system, as shown in Figure 3. When high voltage is delivered to the syringe needle, the suspended polymer solution droplet on the needle tip acquires a high charge density, causing a significant electrostatic repulsion force that disrupts the droplets. The surface tension force of the droplet opposes this force, resulting in the creation of a stable Taylor cone. As the jet travels from the needle tip to the ground collection, viscoelastic elongation causes the diameter of the jet to shrink and the jet to break into smaller jets. The deposition of nanofibers on the ground collector occurs due to solvent evaporation combined with polymer chain entanglement, which result in the deposition of nanofibers on the ground collector [120].

A continuous feeding system is essential for the electrospinning process to obtain long, non-chopped nanofibers uniformly. The feeding system is typically configured with a syringe or peristaltic pump that squeezes a solution or melts of polymers to the needle tip. Although this system is simple, it faces many obstacles when scaling up for higher productivity in industry. There are various ways to maximize system productivity, such as using a multi-needle spinning system or a needless electrospinning setup [121]. Different patterns for the collecting systems, such as rotary drums and discs, have been designed to avoid the drawbacks of classical ground stationary collectors for producing nanofibers for biomedical applications [122,123].

## 7. Photoactive Eluting and Non-Eluting Nanofibers

The chemical structure of the polymer and the fiber diameter significantly influence its photodynamic applications. Additionally, transparent nanofibers are preferred for certain topical photodynamic-mediated antimicrobial effects [124]. The last decade has seen growing interest in the development of nanoscale photoactive antimicrobial materials [125,126,127]. Nanofibers offer a promising solution for the effective delivery of leached PSs to the wound bed, allowing them to reach deeply embedded microbial cells and other biofilm components. For example, El-Khordagui et al. demonstrated the successful loading of MB within Poly(R-3-hydroxybutyrate) nanofibers that were used as PS eluting dressings for accelerated wound healing [128]. In the case of deep microbial colonization and intensive biofilm formation, the elution mode may be preferred to allow for deeper penetration of the PS to dissociate the thick extracellular matrix of the biofilm and reach its recalcitrant microbial cells. It is worth noting that, in addition to its direct antimicrobial effect, PDT could modulate the polarization of M1(inflammatory) and M2 (remodeling) macrophages in the wound bed; this may enhance the healing process in the short term [129]. However, the uncontrolled accumulation of PSs in wound tissue in conjunction with prolonged light exposure can attenuate the wound healing process through the prolonged generation of ROS; this may lead to a prolonged inflammatory phase (M1 accumulation) [130,131]. A stimuli-responsive release of the PSs may be an ideal solution for the on-demand dose-controlled delivery of PS for PDT-mediated wound healing. For instance, a recent study investigated the dual stimuli-responsive release of curcumin from a poly lactic acid nanofibrous membrane covered with ZIF-8/indocyanine green. This nanofibrous membrane was covered by a phase inversion material; it was demonstrated that this membrane exhibited NIR-dependent curcumin release [132].

**Figure 4:**
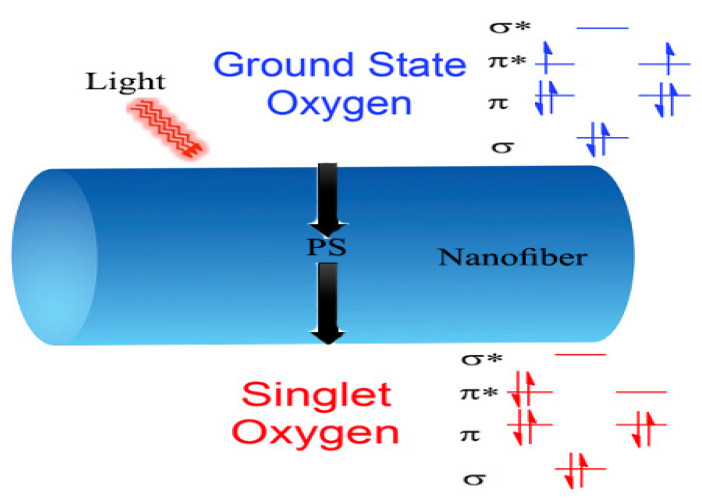
Schematic presentation of the ground state molecular oxygen diffusion inside the polymeric matrix to reach the PS. The energy transfer from PS to the molecular oxygen result in the formation of singlet oxygen with sufficient lifetime to reach nanofiber surface.

In contrast, the photoactive nanofibers that do not elute rely primarily on the production of ^1^O_2_ within the matrix of the nanofiber, which then diffuses to the surface. As a result, their antimicrobial properties are limited to the surface of the nanofibers or near them due to the short lifespan of ^1^O_2_ [133]. Nanofibers are considered excellent photoactive nanoplatforms due to their small diameters, ranging from tens to hundreds of nanometers. As a result, the photogenerated ^1^O_2_ can easily reach the surface of nanofibers, which is ideal for microbial destruction, as shown in Figure 4. Jing Sun et al. aimed to reduce the distance traveled by ROS to reach their targets, and they achieved this by electrospinning MB-loaded mesoporous silica nanoparticles modified by trichloro (1H, 1H, 2H, 2H heptadecafluorodecyl) silane to create ROS generators. They used zein and PCL as a polymeric matrix. Furthermore, the superhydrophobic nature of the fluorinated mesoporous silica nanoparticles allowed for their distribution on the nanofiber’s surface [134]. Another report demonstrated a feasible approach to near-surface PSs through the co-electrospinning of PCL and metal–organic framework (MOF) nanoparticles loaded with RB [135].

The selected polymeric matrices should allow sufficient molecular oxygen diffusion, and no interference with intersystem crossing to reach the PS triplet state. A recent attempt to enhance the delivery of molecular oxygen to diabetic ulcers was studied. Black phosphorus (BP) nanosheets and hemoglobin (Hb) were deposited on electrospun poly-L-lactide nanofibers using quaternized chitosan and hyaluronic acid. BP transformed the NIR radiation into heat and this thermal energy boosted Hb to release oxygen [136]. Furthermore, the materials for nanofibers’ fabrication should be precisely selected, favoring those with the lowest tendency to quench the generated ^1^O_2_ and those exhibiting a rapid transportation ability of ^1^O_2_ to the fiber surface. For example, the successful in situ electrospinning of NaYF_4_:Yb/Tm@NaYF_4_:Nd@Curcumin using PCL as the polymer was reported. The produced photoactive membrane exhibited a potent antimicrobial activity due to its efficient quantum yield of ROS due to its efficient molecular oxygen penetration to reach the loaded PS and its negligible quenching of ^1^O_2_ [137]. Recently, Jun Zhang et al. fabricated a polyvinyl pyrrolidone (PVP)-based nanofibrous dressing with small holes in the nanofiber body (4 nm), ameliorating the release of ROS without leaking the PS-loaded nanoparticles [138].

## 8. Synergic Issues for the Enhanced Antimicrobial Effect of Photoactive Nanofibers

There are numerous strategies to enhance the antimicrobial effect of photoactive nanofibers.

### 8.1. Nanoparticle–Nanofiber Hybrids

PSs directly loaded into nanofibrous mats suffer from uncontrolled PS release. This could be optimized by encapsulating the PS into nanoparticles and in situ electrospinning those nanoparticles into nanofibers. Curcumin was encapsulated in Upconversion nanoparticles (UCNPs) composed of thulium, ytterbium, neodymium, and yttrium. The produced core–shell nanoparticles were mixed with PCL and PVP and electrospun. The produced wound dressing was able to produce ROS upon illumination with 808 nm and a total eradication of MRSA and *E. coli* was achieved [137]. Melanin was employed as a natural PS and it was formulated as nanoparticles that were in situ electrospun with PCL. The synthesized nanofiber responded to irradiation using UV-A light and could be utilized as a drug delivery system for antibiotics [139]. Tetrakis (4-carboxyphenyl) porphyrin was incorporated into MOF and embedded into PCL nanofibers. Irradiating the wound dressing using 630 nm light produced ROS and exerted a potent antimicrobial activity against MRSA and *E. coli* [140]. Another example of a porphyrin PS was meso tetrakis (N-methyl pyridinium-4-yl) porphyrin tetratosylate salt. It was encapsulated into chitosan nanoparticles and the latter were embedded into a poly-polymer blend of polyurethane, PCL, and PEG. The synthesized mat was photoresponsive to 635 nm and irradiation for 30 s was sufficient to exhibit a potent antimicrobial activity and promote wound healing [141]. Furthermore, meso-tetraphenylporphyrin was dopped over silver nanoparticles and embedded into polymethylmethacrylate nanofibers. For this system, irradiation using 405 nm resulted in a complete remediation of *Staphylococcus epidermidis* and *Enterococcus faecalis*. Longer irradiation showed the photostability of the system, suggesting its suitability for PDT-mediated antimicrobial applications [142]. Photoresponsiveness and a mediating of PDT was also reported when carbon quantum dots were embedded in polyacrylonitrile nanofibers. For this system, visible light emitted from a Xe lamp triggered the production of ROS and subsequent antimicrobial activity against various Gram-positive and Gram-negative pathogens [143].

### 8.2. Photoactive and Antimicrobial-Releasing Nanofibers

As previously mentioned, microbial colonization in the form of a biofilm requires aggressive intervention; consequently, nanofibrous dressings may be an ideal nanoplatform. This can be attributed to the possible dual effect of PDT and antibiotic release [144]. Abdelkhalek et al. successfully fabricated cellulose acetate/polyethylene oxide (PEO) nanofibers loaded with MB to mediate PDT-driven biofilm matrix destruction. Additionally, an outer cellulose acetate/PEO/ciprofloxacin layer was added after the PDT session to accelerate the recalcitrant microbial cell destruction. These interventions accelerated the healing of DFU in an animal model [145]. Moreover, a marked enhancement in the antimicrobial effect of PDT with zinc phthalocyanine was observed by loading the PS on silver nanoparticles known for their broad-spectrum antimicrobial activity and embedding these in silica-based nanofibers [146]. Both the antibiotic mitomycin and the PS indocyanine green were loaded into cellulose nanofibers. They were formulated, along with magnetic iron oxide nanoparticles and Pluronic F-127, into a three-dimensional wound dressing. The nanofibrous scaffold was adhesive to the tissues, released both drugs, was inert to fibroblasts, and possessed a profound antibiofilm activity [147]. Benzalkonium chloride, known for its broad-spectrum antimicrobial activity, was co-loaded with porphyrinic MOF (PCN-224) into PCL nanofibers. PCN-224 produced ROS and benzalkonium chloride disrupted microbial lipid molecules, as proven at an in vitro and an in animal model for infected wounds [148].

### 8.3. Photodynamic and Nitric Oxide-Synergistic Antimicrobial Nanofibrous Dressings

Nitric oxide is characterized by its involvement in inflammatory responses, enhanced angiogenesis, and its potent antibiofilm effect. These effects have expanded its applications in wound dressings and tissue scaffolds. Some of these preparations rely on the integration of NO donor molecules such as S-nitrosoglutathione into electrospun nanofibers [149]. A smart NO-releasing nanofibrous membrane was fabricated to utilize the ROS released by PDT for the generation of NO from L-Arginine; as a result NO-assisted PDT antibacterial behavior was achieved [150]. NO could be generated from NIR II PS, characterized by their ability to reach beyond 2.6 cm of depth into skin tissues [151]. Polystyrene nanofibers were functionalized with N-(3-aminopropyl)-3-(trifluoromethyl)-4-nitrobenzenamine, which acted as a NO donor. Furthermore, PS 5,10,15,20-Tetrakis(N-methylpyridinium-4-yl)-porphyrin tetra-p-toluensulfonate (TMPyP) and zinc(II) 2,9,16,23-tetrakis(N methyl-pyridiumoxy)phthalocyanine tetraiodide (ZnPc) were also incorporated into the nanofiber. Interestingly, under visible light illumination, the nanofiber produced ROS and NO. The developed nanosystem could efficiently, in a dual manner, treat *E. coli* infection [152].

### 8.4. Photodynamic/Photothermal Synergy in Nanofibrous Matrices

PDT alone may barely eradicate certain microbial infections; consequently, using a binary model of PDT with a photothermal treatment may provide an ideal solution. Jing Sun et al. fabricated upconverting nanoparticles and titanium oxide (UCNPs@TiO_2_) core–shell nanoparticles as a PS, which was doped with graphene oxide (GO) for photothermal conversion. The nanoparticles were electrospun in a polyvinylidene difluoride (PVDF) nanofibrous matrix. Upon irradiation with NIR light (980 nm), the elevated temperature in conjunction with the released ROS led to broad-spectrum microbial eradication [153]. Researchers have developed nanoparticles that can generate controlled temperatures below 45 °C to protect wound tissues, as extreme elevations in temperature may further deteriorate the tissues [154]. In a recent study, a nanofiber composed of carboxymethyl cellulose was loaded with MoS_2_/TiO_2_ to mediate the photothermal response after irradiation using 808 nm. Simultaneously, the nanofiber was loaded with MB that mediated the PDT upon irradiation with 660 nm. It is worth noting that TiO_2_ exerted oxidase activity. Thus, the developed nanosystem was multimodal and Escherichia coli and Staphylococcus aureus were efficiently treated [155].

## 9. Conclusions

While chronic wounds are imposing a global burden on health, innovations in nanotechnology could show promising results. Combining nanotechnology, in the form of polymeric nanoparticles and nanofibers, with photodynamic therapy can help improve the management of chronic wounds.

## Figures and Tables

**Table 1:** Summary of some studies of chitosan-based nanoparticles loaded with PSs to mediate antimicrobial PDT.

Nanosystem Composition	PS	Major Findings	Ref.
Core–shell nanoparticles of gold coated with chitosan and loaded with PS	Curcumin	- Curcumin release was pH-dependent, with a higher rate under acidic conditions. - Combined photothermal and PDT against *S. aureus* and *E. coli*. - Good biocompatibility was confirmed.	[94]
Chitosan nanoparticles loaded with PS	Emodin	- The highest cellular uptake of tested *Streptococcus mutans* biofilm-forming bacteria took place within 5 min. - Irradiation using blue light decreased bacteria viability and lactic acid production and damaged their DNA.	[95]
Water-soluble magnetic iron oxide nanoparticles coated with chitosan and loaded with PS	Ce6	- Excellent association with MRSA was reported. - Deep penetration of the bacterial biofilm was achieved. - An accelerated wound healing in the tested mouse model and excellent biocompatibility were proven.	[96]
Hollow silica nanoparticles whose pores are covalently anchoring chitosan and coated with PS	Ce6	- Ce6’s loading efficiency was 80% and its release was pH-controlled. - Irradiated nanosystem eradicated the bacteria and destroyed *S. aureus* biofilm in the in vitro and in vivo models. - Regeneration of the infected wound was achieved in 8 days.	[97]
Self-assembly of carboxymethyl chitosan nanoparticles encapsulating the PS	Hematoporphyrin	- Nanosystem was safe for mammalian cells. - Photoquenching was reduced, and ROS produced was higher than the free form of the PS. - 97% bactericidal activity on tested Gram-positive and Gram-negative bacteria was achieved.	[98]
Gold–silver nanoparticles coated with chitosan and loaded with PS	Toluidine blue	- Strong antibiofilm activity against polymicrobial and single-strain biofilms was achieved. - Complete remedy of a type 2 DFU animal model was feasible.	[99]
Iron oxide nanoparticles complexed with the PS and coated with chitosan nanoparticles	Tetrakis (4-carboxyphenyl) porphyrin	- High dispersibility in aqueous medium was achieved. - Good binding to the bacterial cell membrane was proved. - Combined photothermal and PDT antibacterial activity against tested *S. aureus*, *E. coli*, and MRSA was confirmed.	[100]
Chitosan nanoparticles encapsulating the PS	Indocyanine green	- Planktonic and biofilm reduction of *A. baumannii* was achieved after irradiation using 810 nm.	[86]
Carboxymethyl chitosan-polyethyleneimine conjugated with PS	Protoporphyrin IX	- A stable nanosystem for up to 28 days was obtained. - Minimal toxicity to fibroblasts and no hemolysis was reported. - Microbial cell membrane disruption was confirmed.	[101]

## Data Availability

Data are available upon request to the corresponding author.
